# Supplementary material for: Asymmetric topological pumping in nonparaxial photonics
Source: Nat Commun. 2022 Jan 11;13:249. doi: 10.1038/s41467-021-27773-9 (PMC8752818; doi:10.1038/s41467-021-27773-9)
Supplement: Supplementary file 1 — Supplementary Information [file 41467_2021_27773_MOESM1_ESM.pdf]

Supplementary Materials:
Asymmetric topological pumping in nonparaxial photonics

Qingqing Cheng[1], Huaiqiang Wang[2,3], Yongguan Ke[4,5], Tao Chen[1], Ye Yu[1], Yuri S. Kivshar[6], Chaohong Lee[4,5], and Yiming Pan[7]

[1]Shanghai Key Laboratory of Modern Optical System, School of Optical-Electrical and Computer Engineering, University of Shanghai for Science and Technology, Shanghai 200093, China
[2]National Laboratory of Solid State Microstructures, School of Physics, Nanjing University, Nanjing 210093, China
[3]Department of Physics, University of Zurich, 8057 Zurich, Switzerland
[4]Guangdong Provincial Key Laboratory of Quantum Metrology and Sensing & School of Physics and Astronomy, Sun Yat-Sen University (Zhuhai Campus), Zhuhai 519082, China
[5]State Key Laboratory of Optoelectronic Materials and Technologies, Sun Yat-Sen University (Guangzhou Campus), Guangzhou 510275, China
[6]Nonlinear Physics Centre, Research School of Physics, Australian National University, Canberra ACT 2601, Australia
[7]Physics Department and Solid State Institute, Technion, Haifa 32000, Israel

November 25, 2021

Contents

1 Different designs of waveguide structures 2
- 1.1 Control of coupling coefficient . . . 2
- 1.2 Control of propagation constant . . . 2
- 1.3 Design methods for different samples . . . 3

2 Precise coupling coefficient in Rice-Mele model 4

3 Experimental platform 5

4 Comparison between simulation and experiment results 6

5 Direct drivation of negative NNN coupling coefficient 7

6 Deviation of Eq. (3) from Eq. (2) in the main text 8

7 Pade approximations 10

8 Increasing wavevector k in infrared regime 11

9 Decreasing the strength of κ_{NN} 12

10 Different total lengths of waveguide arrays 13

1 Different designs of waveguide structures

1.1 Control of coupling coefficient

We should address the waveguide array configuration to mimic a tight-binding Schrodinger equation in optically coupled waveguides in two aspects: the coupling coefficients (κ) between waveguides and the on-site propagation constants (β) of each waveguide. First, compared with the control of the propagation constant (see the next section), the control of the coupling coefficient profiles is straightforward. Since the coupling strength between two guiding modes exponentially decays with the distance between two waveguides, one can change the coupling coefficients by varying the waveguide spacing. Fig. S1 shows the coupling coefficient as a function of the distance, which indicates that the coupling coefficient can be tuned in a wide range.

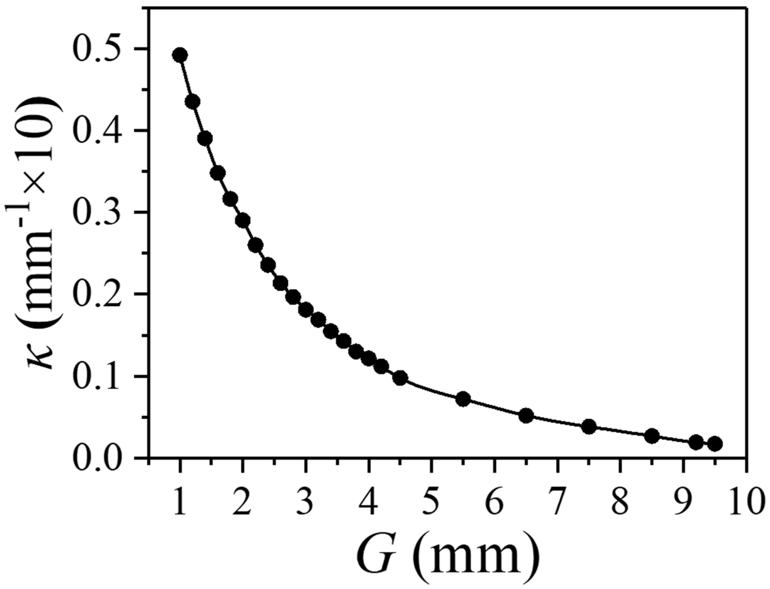

Figure S1: Coupling coefficient as a function of spacing between two waveguides.

1.2 Control of propagation constant

Second, to control the propagation constant, we proposed five types of flexible designs based on "H- bar" units by varying height structure (h), periodic structure (p), width structure (w), up-down shifting structure (s), and rotating structure (a). The existing spoof surface plasmon polaritons (SPP) on the ultrathin corrugated metallic waveguide hold similar dispersion characteristics as SPP in optical frequency, whose dispersion curves are significantly deviated from the light line as shown in Figs. S2(a,b,c,g,h). The dispersion curves are calculated by the finite-difference time-domain CST studio method. Especially in these five types of designs, the dependence of the parameters height (h), width (w), and period (p) in the "H-bar" structure strongly affects the propagation constant (on-site potential) of the eigenmode, as shown in Figs. S2(d,e,f). Hence, we only consider the varying propagation constant via modulation of the height, width, or period parameters in the topological pumping simulation experiments.

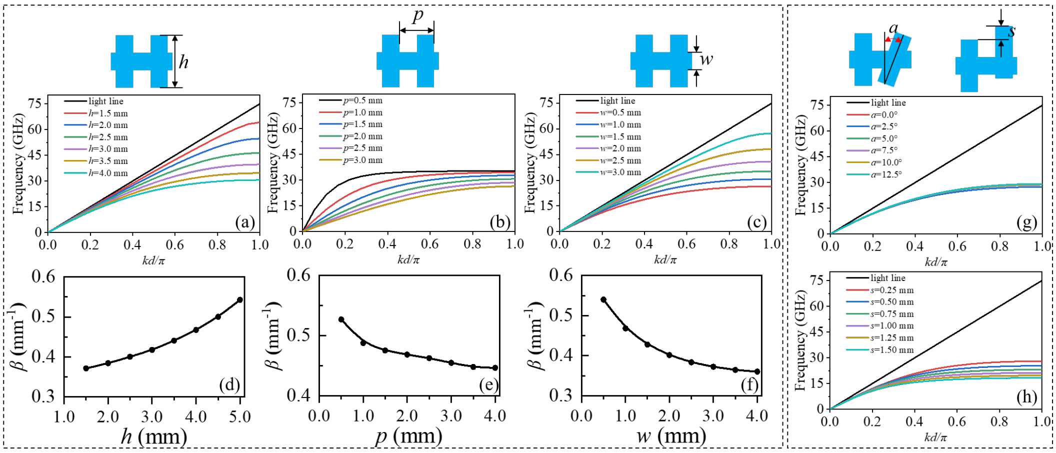

Figure S2: Dispersion curves and propagation constants in different designs of waveguide structures. (a,b,c,g,h) Dispersion curves for varying height structure, periodic structure, width structure, up-down shifting structure, and rotating structure, respectively. (d,e,f) propagation constants as functions of height, period, and width, respectively.

1.3 Design methods for different samples

To match the on-site potential modulation to the sine function of the propagation direction (z), we chose to realize it with the height parameters in the main text. The instantaneous height parameters of the odd-numbered waveguides are selected to be 4 mm ($\varphi = 0$), 3.5 mm ($\varphi = \pi/2$), 4 mm ($\varphi = \pi$), 4.5 mm ($\varphi = 3\pi/2$) and 4 mm ($\varphi = 2\pi$). The on-site potentials of the odd-numbered waveguide satisfy $0.47 - 0.03 \times \sin(2\pi z/42)$. Similarly, we arrange height parameters of the even-numbered waveguides as 4 mm ($\varphi = 0$), 4.5 mm ($\varphi = \pi/2$), 4 mm ($\varphi = \pi$), 3.5 mm ($\varphi = 3\pi/2$) and 4 mm ($\varphi = 2\pi$), and the on-site potentials on the even-numbered waveguide coincide with $0.47 + 0.03 \times \sin(2\pi z/42)$. To modulate the coupling coefficients, the waveguide array with bending in the propagation direction will make the coupling coefficients between the $(2n-1)$th and $2n$th waveguides satisfy $0.34 + 0.21 \times \cos(2\pi z/42)$ as the cosine curve, and the coupling coefficients between the $2n$th and $(2n+1)$th th waveguides will match $0.34 - 0.21 \times \cos(2\pi z/42)$. Finally, we summarize the structural details, the on-site potential, and coupling coefficients, as shown with a partially enlarged view in Fig. S3(a).

While the waveguide spacings are maintained the same to modulate the onsite potentials, we also arranged the structure by varying the width and period of "H-bar" units, as shown in Fig. S3(b) and S3(c), respectively. In the modulation of the period, although the propagation constant departs from the sine function, it only affects the micromotion dynamics of the pumping process. However, it maintains the topological nature of pumping process owing to the topological invariant staying the same.

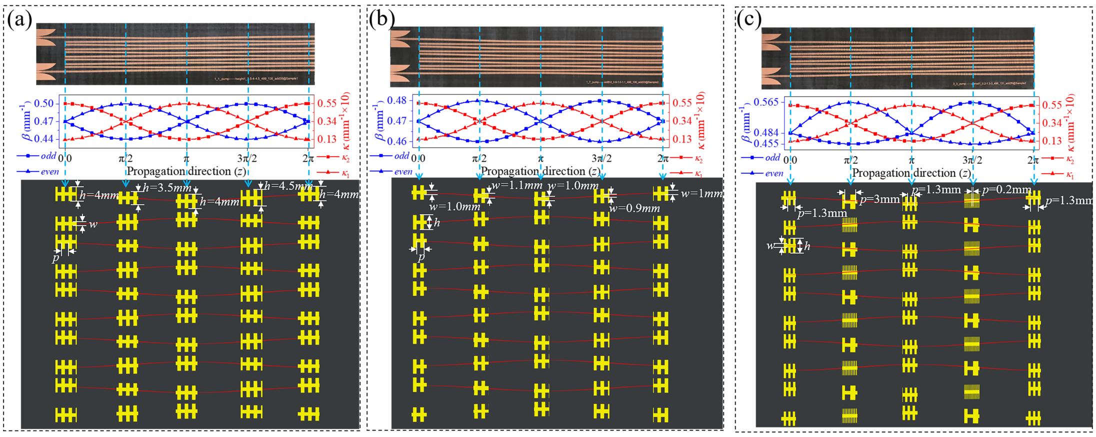

Figure S3: Experimental samples (top), on-site potential energy and hopping strength (middle) and partial enlarged details (bottom) for (a) height-varying design, (b) width-varying design and (c) period-varying design.

2 Precise coupling coefficient in Rice-Mele model

Strictly speaking, the coupling coefficients depend on not only the spacings, but also relate to the geometry of waveguide structures. But in the practical design, the coupling coefficients are usually considered as only depending on the spacing because of the nearly exponential decay with spacing and the slow change with structural parameters. To precisely describe the coupling coefficient, we numerically calculate the coupling coefficients between the first and second waveguides with the same parameters at different eight positions of ϕ=$\pi/8$, $2\pi/8$,...$7\pi/8$ and π in the Rice-Mele model waveguide array. When injecting microwave from one waveguide, the microwave will oscillate between the two waveguides and the coupling coefficient (κ) can be derived from the length (L) of the oscillations by the relation $\kappa \times L = \frac{\pi}{2}$. By comparing the obtained value with the sine function considering only the spacings, we find that there is small difference between the coupling coefficient used in the manuscript and the updated coupling coefficient, as shown by the star marks on the curve of Fig. S4.

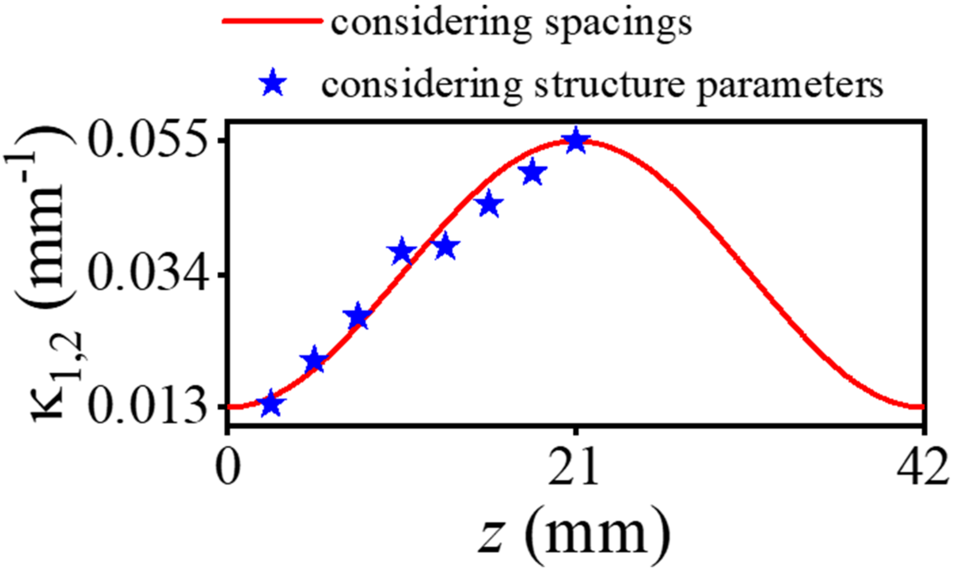

Figure S4: Comparison of coupling coefficients with considering spacings between waveguides (red solid line) and with simultaneously considering both spacings and structure parameters (marked as star).

3 Experimental platform

We carried out experiments utilizing ultrathin bending metallic arrays of coupled "H-shape" waveguides, which support SSPPs at microwave to infrared wavelength. There are three designs for the "H-shaped" waveguide structure to obtain the modulation of the on-site potential. The sample fabricated on a printed circuit board (F4BK) with dielectric constant 2.65, loss tangent 0.001, and thickness 0.2 mm is shown in Fig. S5. We conducted the near-field measurements using a near electric-field platform [see Fig. S5], which is composed of a Vector Network Analyzer (VNA) (E5063A), a monopole antenna as the detector, and a translation stages that can move in the x- and y- directions automatically controlled by a stepper motor. The metallic waveguide is connected to port 1 of the VNA through the SMA to feed the energy, and the end is adhered with an absorber to eliminate reflections. To probe the vertical electric component (E_z) of the SSPPs, we fixed the monopole antenna on top of the metallic waveguide around 1.4 mm and connected it to port 2 of the VNA for recording the field distributions.

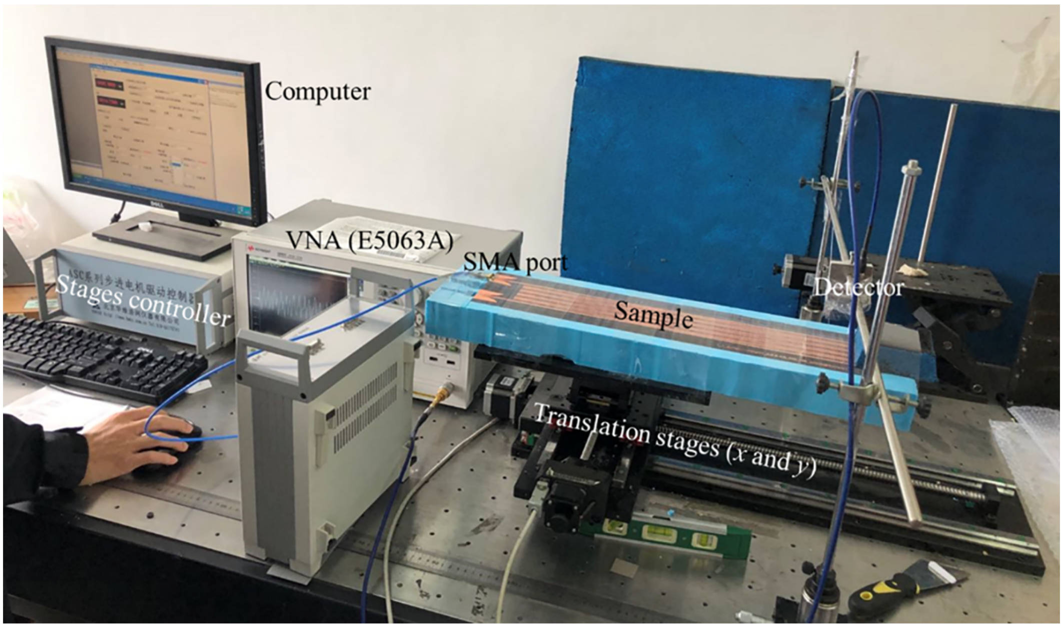

Figure S5: Experimental platform that consists of stages controller, VNA (E5063A), SMA port, sample and translation stages.

4 Comparison between simulation and experiment results

By solving the original non-paraxial Helmholtz equation, we give a direct numerical software (CST Studio) simulations of the light propagation in the three different modulated waveguide arrays, without relying on coupled mode theory under a paraxial approximation. The numerical simulations of light propagation injected from left boundary and right boundary waveguides are respectively shown in left-top and right-top panels of Fig. S6 (a)-(c), corresponding to height-varying, width-varying and period-varying designs. The parameters are chosen as the same as those in the experimental designs. For comparison, we also present the corresponding experiment results in the bottom panels of Fig. S6 (a)-(c). All the numerical simulations agree well with the experimental ones. Comparison with the symmetric topological pumping of light with a much higher frequency ($\lambda = 1.55\mu m$, the corresponding frequency $f = 194$THz) in Fig. 4, these generic asymmetric topological pumping of microwave with a lower frequency 17GHz indicate that the asymmetric behaviors come from non-paraxial effect of microwave.

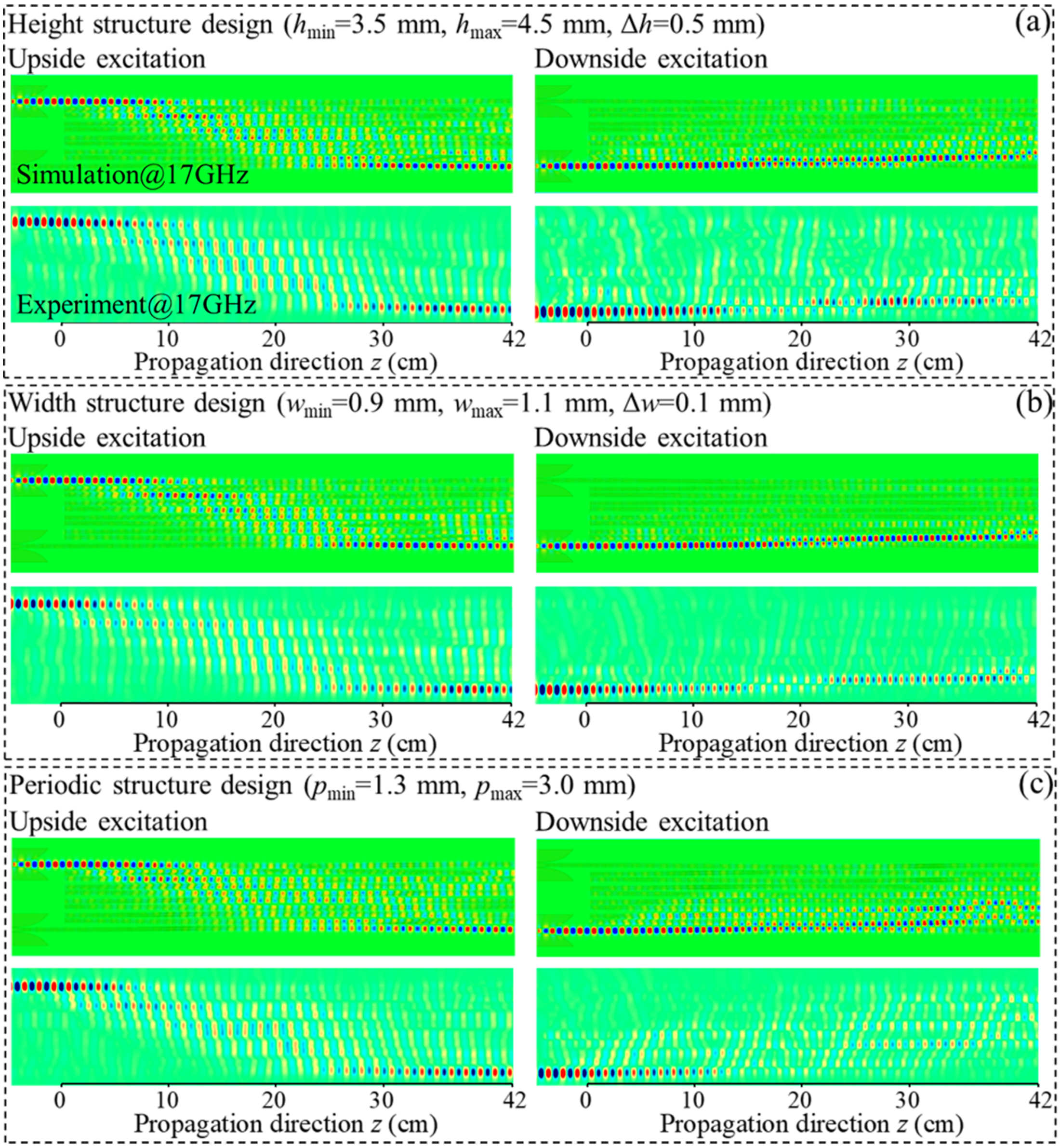

Figure S6: Comparison between numerical simulations of light propagation injected from left boundary (top-left panel) and right boundary (top-right panel) and the corresponding experiment results (bottom panel) for (a) height-varying design, (b) width-varying design and (c) period-varying design.

5 Direct drivation of negative NNN coupling coefficient

From the experimental results, it is not difficult to find that most of the electromagnetic field tunnels to the third waveguide at the position of ϕ=$\pi/2$ when excited from the first waveguide. But most of the electromagnetic field is still on the tenth waveguide at the position of ϕ=$\pi/2$ when excited from the tenth waveguide. We respectively take out the structural parameters of the first, second and third waveguide, as well as the eighth, ninth and tenth waveguide at the position of ϕ=$\pi/2$, and build three straight waveguides structure related to these parameters. Based on the simulation results in three coupled waveguides, we can acquire the negative NNN coupling ($\tilde{\kappa}_{\rm NNN}$) induced by nonparaxiality, as shown in Fig. S7. $\kappa_{eff} = \tilde{\kappa}_{1,3} + \frac{\kappa_{1,2}\kappa_{2,3}}{\beta_1-\beta_2}$ or $\kappa_{eff} = \tilde{\kappa}_{8,10} + \frac{\kappa_{8,9}\kappa_{9,10}}{\beta_8-\beta_9}$ represents the effective coupling coefficient in the three coupled waveguide array, where $\tilde{\kappa}_{1,3} = \kappa_{1,3} + \tilde{\kappa}_{\rm NNN}$, $\tilde{\kappa}_{8,10} = \kappa_{8,10} + \tilde{\kappa}_{\rm NNN}$, $\kappa_{1,3} \simeq 0.0019mm^{-1}$ (the spacing between the first and third waveguides is 9.67 mm), $\kappa_{8,10} \simeq 0.0028mm^{-1}$ (the spacing between the eight and tenth waveguides is 8.7 mm), $\kappa_{1,2} = \kappa_{2,3} = \kappa_{8,9} = \kappa_{9,10} \simeq 0.021mm^{-1}$, $\beta_1 = \beta_3 = \beta_9 \simeq 0.44mm^{-1}$ and $\beta_2 = \beta_8 = \beta_{10} \simeq 0.5mm^{-1}$. The effective coupling length L_{eff} of the two kinds of three coupled waveguides are extracted as $135mm$ and $440mm$, respectively. From the relation $\kappa_{eff} \times L_{eff} = \frac{\pi}{2}$, we can easily calculate κ_{eff} to be -0.01164m^{-1} and 0.00357m^{-1}, respectively. Attention should be payed to the sign of κ_{eff}, otherwise the opposite results will be obtained, that are, coupling phenomenon is suppressed in the first-second-third waveguide and enhanced in the eighth-ninth-tenth waveguide structures. By using one of the κ_{eff}, $\tilde{\kappa}_{\rm NNN}$ turns out to be $\tilde{\kappa}_{\rm NNN} = -0.00619mm^{-1}$ or $\tilde{\kappa}_{\rm NNN} = -0.00658mm^{-1}$, in agreement with the our fitting value.

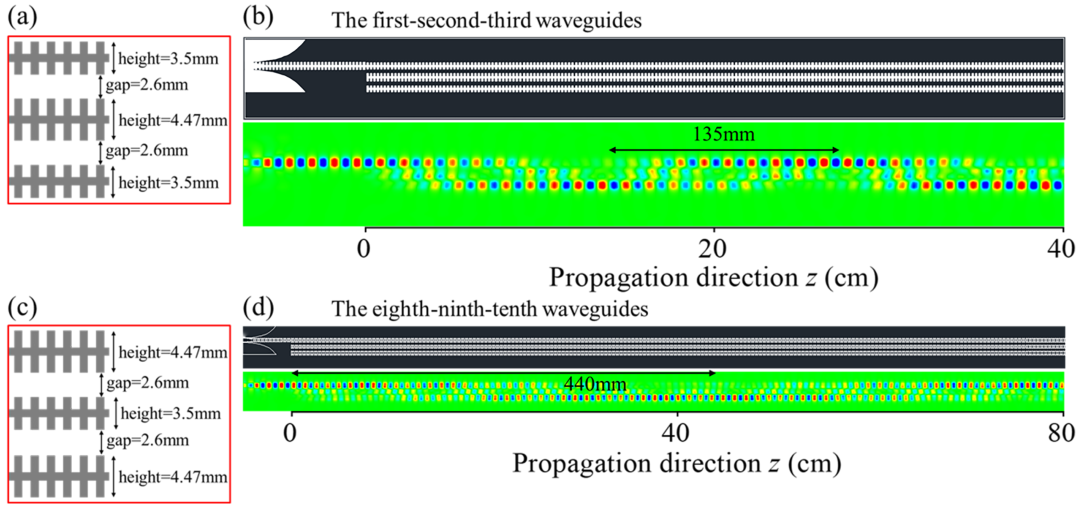

Figure S7: Top: (a) Schematic diagram of three straight waveguides with the same parameters as the first-second-third waveguides extracted at the position of ϕ=$\pi/2$ in Rice-Mele modeled waveguide array. (b) Simulation results of microwave propagating in the three waveguide structures. Bottom: (c) Schematic diagram of three straight waveguides with the same parameters as the eighth-ninth-tenth waveguides at the position of ϕ=$\pi/2$. (d) Simulation results of microwave propagating in the coupled three waveguide structures.

6 Deviation of Eq. (3) from Eq. (2) in the main text

The electromagnetic waves are confined on the surface (y direction) of the microwave waveguides, hence we can start from the two-dimensional Helmholtz equation for the (x,z)-plane monochromatic electromagnetic waves,

$$\nabla^2 E(x,z;t) + k^2 n^2(x,z) E(x,z;t) = 0, \tag{1}$$

Here, k is the wave vector, and the refractive index $n(x,z)$ is designed according to the Rice-Mele type model. Substituting the in-plane electromagnetic field $E(x,z;t) = \psi(x,z)e^{i\omega t - ikn_0 z}$ into the Helmholtz equation, the above equation can be simplified as

$$\frac{\partial^2\psi(x,z)}{\partial x^2} + \left(\frac{\partial^2\psi(x,z)}{\partial z^2} - 2ikn_0\frac{\partial\psi(x,z)}{\partial z} - k^2 n_0^2\psi\right) + k^2 n^2(x,z)\psi(x,z) = 0 \tag{2}$$

The above formula can be written as Eq. (2) in the main text,

$$i\frac{\partial\psi}{\partial z} - \frac{1}{2kn_0}\frac{\partial^2\psi}{\partial z^2} = H\psi, \tag{3}$$

$$\Rightarrow \left(1 + \frac{i}{2kn_0}\frac{\partial}{\partial z}\right) i\frac{\partial\psi}{\partial z} = H\psi. \tag{4}$$

$$\Rightarrow i\frac{\partial\psi}{\partial z} = \left(1 + \frac{i}{2kn_0}\frac{\partial}{\partial z}\right)^{-1} H\psi \equiv \frac{H}{1 + \frac{i}{2kn_0}\frac{\partial}{\partial z}}\psi, \tag{5}$$

in which $H = \frac{1}{2kn_0}\frac{\partial^2}{\partial x^2} + \frac{k}{2}\left(\frac{n^2 - n_0^2}{n_0}\right)$. Next, we replace the Helmholtz-Hamiltonian operator by the structurally modelled Rice-Mele Hamiltinian H_{RM} in our system. Along the transverse direction, we can expand the electromagnetic field in the basis of Wannier functions $w_j(x,z)$ as,

$$\psi(x,z) = \sum_j \varphi_j(z) w_j(x,z), \tag{6}$$

where the Wannier functions $w_j(x,z)$ are real localized functions centered at the jth waveguides, satisfying the orthogonal relation

$$\int w_j(x,z) w_l(x,z) dx = \delta_{j,l}. \tag{7}$$

Substituting Eq. (7) into Eq. (3), we can obtain

$$\begin{aligned}&\sum_j i\frac{\partial\varphi_j(z)}{\partial z} w_j(x,z) + \sum_j \varphi_j(z) i\frac{\partial w_j(x,z)}{\partial z}\\ &-\frac{1}{2kn_0}\sum_j\left(w_j(x,z)\frac{\partial^2\varphi_j(z)}{\partial z^2} + \varphi_j(z)\frac{\partial^2 w_j(x,z)}{\partial z^2} + 2\frac{\partial\varphi_j(z)}{\partial z}\frac{\partial w_j(x,z)}{\partial z}\right)\\ &= \sum_j H\varphi_j(z) w_j(x,z)\end{aligned} \tag{8}$$

Projecting the above equation into Wannier functions $w_l(x,z)$ and integrating over x, we can obtain

$$\begin{aligned}&i\frac{\partial}{\partial z}\varphi_l(z) + \sum_j \varphi_j(z)\int w_l(x,z) i\frac{\partial}{\partial z} w_j(x,z) dz\\ &-\frac{1}{2kn_0}\frac{\partial^2}{\partial z^2}\varphi_l(z) - \frac{1}{2kn_0}\sum_j\left(\varphi_j(z)\int w_l(x,z)\frac{\partial^2}{\partial z^2} w_j(x,z) + 2\frac{\partial}{\partial z}\varphi_j(z)\int w_l(x,z)\frac{\partial}{\partial z} w_j(x,z)\right)\\ &= \sum_j\int dx\, w_l(x,z) H w_j(x,z)\varphi_j(z)\end{aligned} \tag{9}$$

Here, we need to evaluate the magnitudes of $\int w_l(x,z)\frac{\partial^2}{\partial z^2}w_j(x,z)$ and $\int w_l(x,z)\frac{\partial}{\partial z}w_j(x,z)$. For the waveguides designed as Rice-Mele type model, we can approximate Wannier functions as localized Gaussian functions

$$w_j(x,z) = \frac{1}{\sigma^{1/2}\pi^{1/4}} \exp\left[-\frac{(x-X_j(x,z))^2}{2\sigma^2}\right]. \tag{10}$$

Here, $X_j(x,z) = jd + \nu d\cos(\pi j + 2\pi z/\Lambda)$ are the positions of the jth waveguides. ν is the modulation strength of waveguide spacings with an average value d. σ is the width of Gaussian function whose dependence on z can be effectively neglected. For the $\int w_l(x,z)\frac{\partial}{\partial z}w_j(x,z)$ term, we have

$$\int w_l(x,z)\frac{\partial}{\partial z}w_j(x,z) \approx -\delta_{j,l}\frac{1}{\sigma\sqrt{\pi}}\frac{\nu d\sin(\pi j + 2\pi z/\Lambda)2\pi}{\Lambda\sigma^2}\int \mathrm{e}^{-\frac{X^2}{\sigma^2}}XdX = 0. \tag{11}$$

For the $\int w_l(x,z)\frac{\partial}{\partial z}w_j(x,z)$ term, we have

$$\begin{aligned}
& \int w_l(x,z)\frac{\partial^2}{\partial z^2}w_j(x,z)dx \\
\approx\ & \delta_{j,l}\frac{(\nu d)^2\sin^2(j\pi + 2\pi z/\Lambda)(2\pi)^2}{\sigma^2\Lambda^2}\left(-\frac{1}{\sigma\sqrt{\pi}}\int \mathrm{e}^{-\frac{X^2}{\sigma^2}}dX + \frac{1}{\sigma\sqrt{\pi}}\int\frac{X^2}{\sigma^2}\mathrm{e}^{-\frac{X^2}{\sigma^2}}dX\right) \\
=\ & -\delta_{j,l}\frac{(\nu d)^2\sin^2(2\pi z/\Lambda)(2\pi)^2}{2\sigma^2\Lambda^2}.
\end{aligned} \tag{12}$$

This term does not depend on the waveguides and contributes to an overall shift which can be neglected. At last, we can obtain a model that describes the coupling between φ_j,

$$i\frac{\partial}{\partial z}\varphi_l(z) - \frac{1}{2kn_0}\frac{\partial^2}{\partial z^2}\varphi_l(z) = \sum_j\int dx w_l(x,z)Hw_j(x,z)\varphi_j(z). \tag{13}$$

Here, $\int dx w_l(x,z)Hw_j(x,z)$ are the matrix elements of the Helmholtz-Hamiltonian operator by the structurally modelled Rice-Mele Hamiltinian H_{RM}. The above equation can be further rewritten as

$$i\frac{\partial}{\partial z}\varphi - \frac{1}{2kn_0}\frac{\partial^2}{\partial z^2}\varphi \quad = \quad i\frac{\partial}{\partial z}\left(1 + \frac{i}{2kn_0}\frac{\partial}{\partial z}\right)\varphi = H_{RM}\varphi \tag{14}$$

Then we derive the effective Schrödinger-type form in the main text

$$i\frac{\partial}{\partial z}\varphi = \frac{H_{RM}}{1 + \frac{1}{2kn_0}i\frac{\partial}{\partial z}}\varphi \tag{15}$$

This equation can be iteratively solved to yield the nonparaxial beam propagation of the electromagnetic field.

7 Pade approximations

In the conventional Schrödinger-type equation, $i\frac{\partial}{\partial z}$ is directly given by H_{RM}. However, in our effective Schrödinger-type formula [Eq. (3) in the main text], the operation $i\frac{\partial}{\partial z}$ is related to itself. Such equation can be solved in a self-consistent way, that is, we iteratively substitute $i\frac{\partial}{\partial z}$ in the denominator by $i\frac{\partial}{\partial z}$ and use the initial condition $\left(i\frac{\partial}{\partial z}\right)_{-1} = 0$. For example, the zeroth-order of $i\frac{\partial}{\partial z}$ is given by

$$\left(i\frac{\partial}{\partial z}\right)_0 \approx \frac{H_{RM}}{1+\frac{1}{2kn_0}\left(i\frac{\partial}{\partial z}\right)_{-1}} = H_{RM}, \tag{16}$$

the first-order of $i\frac{\partial}{\partial z}$ is given by

$$\left(i\frac{\partial}{\partial z}\right)_1 \approx \frac{H_{RM}}{1+\frac{1}{2kn_0}(i\frac{\partial}{\partial z})_0} = \frac{H_{RM}}{1+\frac{1}{2k_0 n}\frac{H_{RM}}{1+\frac{1}{2kn_0}\left(i\frac{\partial}{\partial z}\right)_{-1}}} = \frac{H_{RM}}{1+\frac{H_{RM}}{2kn_0}}, \tag{17}$$

and the second-order of $i\frac{\partial}{\partial z}$ is given by

$$\left(i\frac{\partial}{\partial z}\right)_2 \approx \frac{H_{RM}}{1+\frac{1}{2kn_0}(i\frac{\partial}{\partial z})_1} = \frac{H_{RM}+\frac{H_{RM}^2}{2kn_0}}{1+\frac{H_{RM}}{kn_0}}. \tag{18}$$

The right-hand side of the above equations can be written as an effective Hamiltonian $H_{eff}^{n,m} = N(n)/D(m)$ where $N(n)$ and $D(m)$ are polynomials of H_{RM}, which are called as Padé approximants. The first few orders of Padé approximant are given in the Table 1 for details.

The effective Hamiltonians $H_{eff}^{n,m}$ are still Hermitian. Here, we give a simple mathematical proof. We can expand the Hermitian Rice-Mele Hamiltonian as $H_{RM} = \sum_j \kappa_j |\psi_j\rangle\langle\psi_j|$, where κ_j are real and $|\psi_j\rangle\langle\psi_j|$ are Hermitian. The effective Hamiltonian as a real function of Rice-Mele Hamiltonian, $H_{eff} = f(H_{RM})$, can be written as $H_{eff} = \sum_j f(\kappa_j)|\psi_j\rangle\langle\psi_j|$. Because $f(\kappa_j)$ are real and $|\psi_j\rangle\langle\psi_j|$ are Hermitian, the effective Hamiltonian H_{eff} is also Hermitian.

By numerical examination of the matrix element of the (1,0) Padé approximant, we find that the next-nearest-neighboring (NNN) coupling strengths are always negative. To give a comprehensive understanding, for the (1,0) Padé approximant, we make a Taylor expansion of $1/(1 + H_{RM}/(2kn_0))$ around $H_{RM} = 0$ up to the first order, and give an approximation of the effective Hamiltonian,

$$H_{eff} = H_{RM} - \frac{1}{2kn_0}H_{RM}^2, \tag{19}$$

The term $-\frac{1}{2kn_0}H_{RM}^2$ contributes an effective negative NNN coupling which is approximated as $\kappa_{NNN} \approx -\kappa_{NN}^2/2kn_0$. From Eq. (19), we can immediately know that H_{eff} is Hermitian, consistent with the general conclusion mentioned above.

(m,n)	$N(m)/D(n)$
$(1,0)$	H_{RM}
$(1,1)$	$\frac{H_{RM}}{1+\frac{H_{RM}}{2kn_0}}$
$(2,1)$	$\frac{H_{RM}+\frac{H_{RM}^2}{2kn_0}}{1+\frac{H_{RM}}{kn_0}}$
$(2,2)$	$\frac{H_{RM}+\frac{H_{RM}^2}{kn_0}}{1+\frac{3H_{RM}}{2kn_0}+\frac{H_{RM}^2}{4(kn_0)^2}}$
$(3,2)$	$\frac{H_{RM}+\frac{3H_{RM}^2}{2kn_0}+\frac{H_{RM}^3}{4(kn_0)^2}}{1+\frac{2H_{RM}}{kn_0}+\frac{3H_{RM}^2}{4(kn_0)^2}}$

8 Increasing wavevector k in infrared regime

We presented the simulation results of the optical analog of the pumping process based on the similar design, with a much higher frequency ($\lambda = 1.55\mu$m) of the propagating electromagnetic wave. In our curved planar silicon waveguide array placed in vacuum with the amplitude value 30 nm, we choose the thickness as 260 nm ($\varphi = 0$), 210 nm ($\varphi = \pi/2$), 260 nm ($\varphi = \pi$), 310 nm ($\varphi = 3\pi/2$) and 260 nm ($\varphi = 2\pi$). As shown in Fig. S8, the total length of the coupled waveguide array is 500μm, in which the maximum coupling spacing and the minimum coupling spacing are 125 nm and 265 nm, respectively. For the modes existing in the waveguide, we choose the lowest index transverse magnetic (TM0) mode. The range of propagation constant (on-site potential energy) and coupling coefficient (hopping strength) corresponding to the set parameters are $1.464k_0 \sim 2.514k_0$ and $0.0713\mu\text{m}^{-1} \sim 0.345\mu\text{m}^{-1}$, respectively. From the spatial distributions of light field injected from the left and right boundary waveguides, we can deduce that the light injected from left boundary waveguide is almost transferred to the right boundary waveguide, and the reversal pumping process is symmetric to the previous one and also successful.

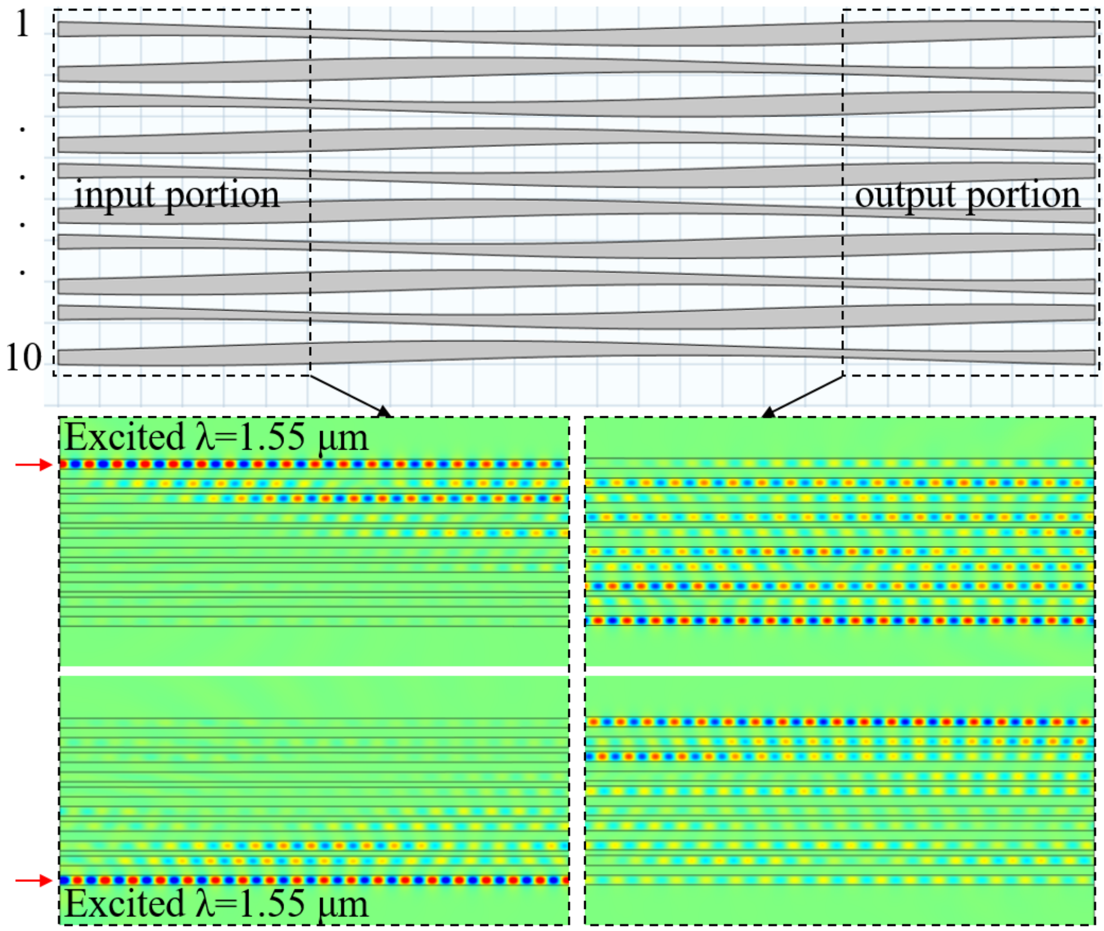

Figure S8: Planar waveguide array and electromagnetic component of TM0 distributions in the coupled waveguide array. The spatial TM0 distribution of the input and output portions indicate that the left-to-right pumping is symmetric to the right-to-left pumping.

9 Decreasing the strength of κ_{NN}

We increase the spacing between the waveguides to decrease the nearest-neighbor coupling κ_{NN}. Four cases were compared in the simulation results: (a) g_{min}=1mm and g_{max}=4.2 mm, (b) g_{min}=2mm and g_{max}=5.2mm, (c) g_{min}=3 mm and (d) g_{max} =6.2mm, g_{min}=4 mm and g_{max}=7.2mm. We find that the asymmetry behavior becomes weaker as the waveguide spacing increases. This is because the decrease of nearest-neighbor coupling leads to the dramatical decrease of next nearest-neighbor coupling, consistent with the theoretical prediction ($\kappa_{NNN} \approx -\kappa_{NN}^2/(2kn_0)$).

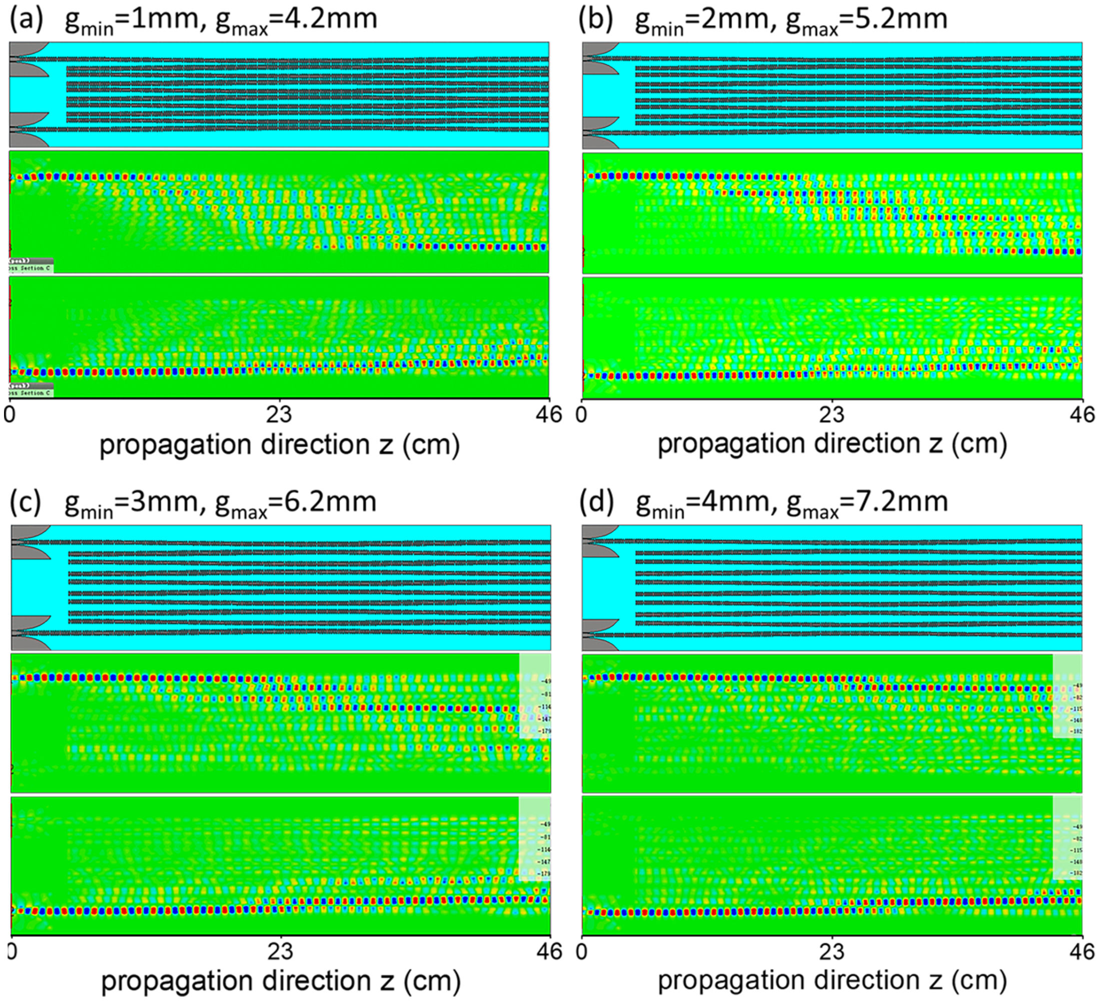

Figure S9: With the decrease of the nearest-neighbor coupling coefficient, the simulation results compare the light beam propagation under the four parameters. (a) g_{min}=1mm and g_{max}=4.2 mm. (b) g_{min}=2mm and g_{max}=5.2mm. (c) g_{min}=3 mm and g_{max} =6.2mm. (d) g_{min}=4 mm and g_{max}=7.2mm.

10 Different total lengths of waveguide arrays

To exclude the nonadiabatic driving as the reason for the asymmetry, we have performed simulations of three Rice-Mele waveguide arrays with increased total lengths, 600mm, and 800mm, respectively. Since the total length plays the role of the modulation period T in the Rice-Mele model, a larger total length corresponds to a smaller modulation frequency, thus rendering the system more "adiabatic". As shown in Fig. S10, asymmetric pumping can be clearly seen in all of the two simulation results. As a result, we can reasonably rule out the nonadiabatic driving as an underlying factor for the asymmetry.

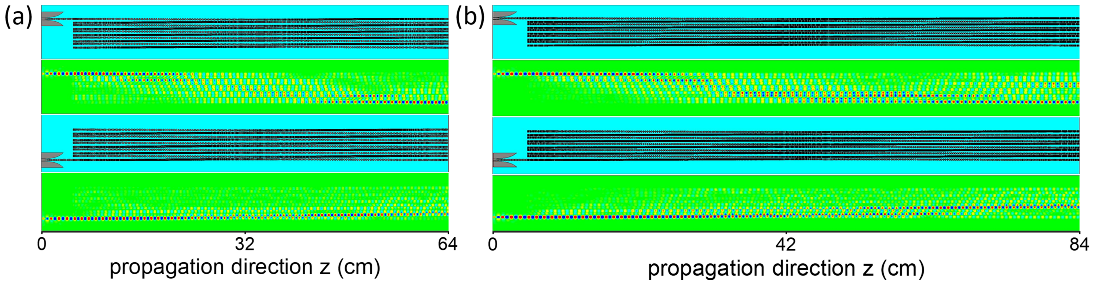

Figure S10: Rice-Mele waveguides arrays with increased total length in the propagation direction (z), namely, (a) 60cm, and (b) 80cm. Note that asymmetric pumping can be clearly seen in all two cases.
